# Household attributes associated with peak period domestic appliance loads

**DOI:** 10.1016/j.heliyon.2021.e07559

**Published:** 2021-07-12

**Authors:** John Curtis

**Affiliations:** a Economic and Social Research Institute, Sir John Rogerson's Quay, Dublin, Ireland; b Trinity College Dublin, Dublin, Ireland

**Keywords:** Electricity, Residential, Appliances, Load shifting, Load curtailment, Customer load profile

## Abstract

Household appliances represent substantial electricity load within the residential sector, particularly during the electricity system's period of peak evening load. While there is broad understanding of the factors that systematically impact on aggregate residential loads, much less is known about appliance loads. A research priority is understanding how socio-demographic, dwelling, and appliance factors are associated with the timing and scale of appliance loads. Using data from Ireland the analysis finds that the number of household occupants; number of appliances; and daytime occupancy of the home are closely associated with appliance loads but varies depending on the time of day. No association is found between appliance uses and building tenure, type or age; or socio-demographic variables such as income, age or education. The empirical findings have relevance for modelling residential electricity loads, and for design of measures to shift residential loads away from the evening peak period.

## 1 Introduction

The management of peak electricity loads and associated operational and environmental benefits has been extensively researched [1], [2]. In recent years the integration of intermittent renewable generation in power systems within the context of climate policy ambition to reduce greenhouse gas emissions has been an important research motivation [3], [4]. Peak load shaving and peak load shifting are specific areas of interest, both in residential and industrial settings. Within the residential sector a number of approaches to demand side management (DSM) are actively pursued, including autonomous control of specific loads, usually related to heating and cooling [5], [6]; and the scheduling of domestic appliances, such as washing machines and dishwashers [7], [8]. To efficiently design and deploy DSM mechanisms, such as load scheduling or autonomous control, requires knowledge of potentially curtailable loads, preferably with spatial granularity, and customers' preferences for electricity services, including their tolerance with respect to service interruption (i.e. delayed heating, cooling, or appliance use). Evening peak load, as individuals return home from work, are a feature of power systems and easily forecast at system level. There is considerable heterogeneity in loads across households, an understanding of which is necessary to develop efficient DSM mechanisms. Research on customer preferences for electricity demand management is ongoing in many electricity markets [9], [10]. As data on customer load profiles is not always readily available, a wide variety of approaches have been developed to construct residential load profiles, including load curve models, stochastic processes and Markov chains [11], [12], [13], [14], [15], [16]. A particular challenge for developing reliable load profile data relates to appliance ownership and use. Carlson et al. [17] note that estimating the number of appliances that contribute to a home's electricity consumption is a difficult, complex problem. Research to quantify the contribution of domestic appliance loads during the evening peak falls roughly into two categories. One might be characterised as an engineering approach, using various methods to identify appliance load profiles among representative households without much consideration to within household determinants of appliance loads [18], [19], [20]. Or alternatively providing an assessment of the demand response potential of large household appliances [21], [22], [23]. For instance, Pipattanasomporn et al. [21] conclude that clothes dryers have the greatest demand response potential, followed by water heaters, air conditioners, dishwashers, clothes washers, refrigerators and finally electric ovens ranked as having no demand response potential. The second category of research on domestic appliance loads has a socio-demographic focus, attempting to understand which dwelling or occupant characteristics are associated with appliance use. In a literature review of factors that systematically impact on total residential electricity demand, Jones et al. [24] identify three key determinants: (1) socio-economic factors (e.g. number of occupants, teenagers, income), (2) dwelling factors (e.g. dwelling age, size), and (3) appliance factors (e.g. number and type of electricity appliances). In a DSM context, a research priority is understanding how these socio-economic, dwelling, and appliance factors impact on the timing of residential loads, in general, and specifically appliance loads that can potentially be shifted to off-peak periods. Several studies have examined determinants of residential appliance loads. For example, in Japan, Matsumoto [25] find that family structure and economic status systematically determine appliance usage. The presence of teenagers increases air conditioner and dishwasher use, while high-income households use appliances such as air conditioners less intensively than low-income households, as they spend more time outside the home. In the United States, Kavousian et al. [26] find that the number of household occupants and appliances best explain daily maximum load, with no significant correlation between electricity consumption and income level, home ownership, or dwelling age. Little attention has focused on the factors that impact the timing of residential appliance loads, with studies by Cetin et al. [27] and Yilmaz et al. [16] being notable exceptions. Cetin et al. [27] study 1-minute resolution load data for four household appliances (i.e. refrigerator, washing machine, tumble dryer, and dishwasher) as well as building and occupant attribute data from 40 homes in Austin, Texas. The methodological approach entails examining the percent of daily energy use load within each hour, segmented across specific use profiles (e.g. weekdays versus weekends, working from home versus not working from home, etc.). Among their findings are that the timing and usage of appliances that require users to initiate use (e.g. washing machines, dishwashers, and tumble dryers) vary greatly between houses compared to appliances that do not require users to manually initiate use (e.g. refrigerator). In households where someone works from home, energy use by appliances is up to 28% greater during normal business hours (09:00–17:00) compared to households where nobody works from home. Washing machines and tumble dryers are the appliances whose energy use profiles are most influenced by whether somebody works from home. Yilmaz et al. [16] find that there is significant variation between households in the number of appliance uses and also between appliance types but their analysis did not consider variations based on occupant attributes.

The purpose of this study is to show how socio-demographic factors, occupancy and appliance ownership affect appliance usage during the evening peak. Amongst the papers cited, this study is closest to Cetin et al. [27], thought the methodological approaches in the two papers are quite distinct. This paper provides estimates of how the number of appliance use cycles in the evening peak (and more generally) differ depending on occupancy levels, whether the dwelling is occupied during the daytime, the number of large domestic appliances, as well as, other socio-demographic characteristics. This has practical relevance for several reasons. An improved understanding of the impact of these factors would be helpful for DSM practitioners, whether in policy development or the design of specific measures to shift residential loads away from the evening peak period. It is also beneficial for residential building energy modelling. While the empirical analysis pertains to the Irish electricity market, the results may have relevance to a wider geographical area.

## 2 Methodology

### 2.1 Data

The primary variable of interest in this analysis is the number appliance use cycles during the evening, coinciding with the peak electricity load period, as well as, across all periods of the day. The survey used to collect the data used here was also used in a previous study by Curtis et al. [28] on preferences for domestic appliance curtailment contracts. Data collection for the analysis was via online survey of the Irish adult population, with a sample drawn from the panel book of a professional survey company. The sample was stratified by geographic location (NUTS III region), gender, age and employment status to match the 2016 Irish Census of Population returns for adults aged 18 and over. The survey was administered in the summer of 2018. Panel members were paid for their participation subject to completion of the survey, which is the standard operating model of the survey company for surveys based on its panel. Two logic questions were incorporated in the survey to screen out respondents not paying adequate attention to the survey questions. A total of 1,080 respondents passed the screening. The questionnaire comprised four sections, the first eliciting respondents' time-specific appliance use habits, the second and third components related to a choice experiment, and the last part collected socio-demographic information. The minimum time necessary to read through the entire questionnaire for a respondent with reasonable literacy skills is 10–12 minutes. Respondents with a survey completion time less than 10 minutes, which totalled 229 people, were excluded from this analysis for the reason that insufficient attention was given to the task. Excessively long completion times are problematic for the same reason. The longest completion time exceeded 7 days, which suggests in that instance the survey was likely completed across multiple days. Observations where completion time exceeded 60 minutes were also excluded. The final sample comprises 812 respondents with a median survey completion time of 15 minutes and a mean of 17.3 minutes.

**Figure 1:**
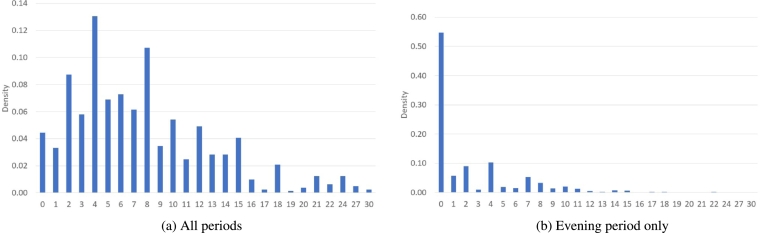
Number of appliance uses per week.

The survey collected information on the number and time period of use for electric ovens, dishwashers, washing machines, and tumble dryers. The analysis here focuses on the latter three appliances, as the demand response potential of electric ovens is considered to be quite low [21]. As the data elicited is based on respondent recall, rather than metered usage, the data is deemed to reflect average or typical usage within a home. The primary variable of interest is the number appliance use cycles per week across all three appliances: dishwashers, washing machines, and tumble dryers. Respondents were also asked to indicate whether the appliance was used during the morning, afternoon, evening or night. While this collection method may not have the accuracy level associated with methods such as smart meter data, it does have the advantage of allowing for the collection of a demographically representative sample including data on occupants characteristics, which are key to understanding the role of socio-demographic factors in appliance use patterns. While smart meters offer the potential to collect precise appliance load data, usually smart meter data contains relatively little, if any, information about the household and its occupants unless supplemented with surveys or register data providing information similar to that used in this study. The analysis here examines the number of appliance uses per week within the evening period, as well as, across all time periods, histograms of which are presented in Fig. 1. While the analysis considers the appliances collectively, the appliance used most frequently is the washing machine, followed by dishwasher and clothes dryers. The correlation coefficient between washing machine and clothes dryer use is 0.48 and slightly greater than 0.3 in the other cases. The correlation coefficient between appliance use frequency, either for individual appliances or in aggregate, and number of household occupants is between 0.28–0.47, whereas for other occupant characteristics the correlation coefficient is substantially lower. Descriptive statistics for the final dataset sample are reported in Table 1.

**Table 1:** Descriptive statistics for variables used in regression analysis (N=812).

Variable	Type ^†^	Mean	Std. Dev.
Number of appliance uses per week	C	7.632	5.479
Number of appliance uses per week mostly during evening peak	C	2.404	3.745
Highest Educational attainment			
Less than full secondary level education	D	0.055	0.229
Secondary level education	D	0.379	0.486
Undergraduate level	D	0.411	0.492
Post-graduate level	D	0.154	0.361
In employment	D	0.562	0.496
Age			
Age 18–34	D	0.244	0.430
Age 35–54	D	0.385	0.487
Age 55+	D	0.371	0.483
Monthly household after tax income			
€0–1999	D	0.265	0.441
€2,000-3999	D	0.381	0.486
€4000+	D	0.196	0.397
item non-response	D	0.158	0.365
Persons living in the household			
1 person	D	0.142	0.349
2 persons	D	0.383	0.486
3 persons	D	0.193	0.395
4 persons	D	0.169	0.375
5+ persons	D	0.113	0.317
If person(s) usually at home during day	D	0.784	0.411
Number of appliances in household (washing machine, dishwasher or clothes dryer)			
0 appliances	D	0.007	0.086
1 appliance	D	0.161	0.368
2 appliances	D	0.358	0.480
3 appliances	D	0.473	0.500

† C=count variable (0,1,2,3, …), D=categorical variable (Equal to 1 if true, 0 otherwise).

### 2.2 Modelling

The variable of interest measures the number of appliance uses per week (including during the evening peak period) and comprises non-negative integers, i.e. a count variable. Poisson and negative binomial regression models are typically used to analyse such data. Count variables are usually right skewed, as is the case with the dataset under consideration, plus have a variance that increases with the mean of the distribution. A feature of the Poisson distribution is that its mean equals its variance, which is uncommon in real data. The negative binomial distribution does not assume such a relationship and is therefore used for the current analysis. Two separate negative binomial models are used to analyse the data, as the data generating process for the number of appliance uses per week differs depending on whether it covers just the evening peak electricity load period or across all periods of the day.

For the number of appliance uses per week across all periods of the day, the standard negative binomial regression model is utilised, and which is also termed the NB2 model. If $y_{i}$ is the number of appliance uses per week by household *i*, the negative binomial probability distribution is

(1) $$f(y;\mu ,\alpha )=\frac{\Gamma (y_{i}+\alpha^{-1})}{\Gamma (y_{i}+1)\Gamma (\alpha^{-1})}{\left(\frac{1}{1+\alpha \;exp(x_{i}\beta )}\right)}^{\frac{1}{\alpha }}{\left(\frac{\alpha \;exp(x_{i}\beta )}{1+\alpha \;exp(x_{i}\beta )}\right)}^{y_{i}}$$

where the relationship between the fitted mean of the model, *μ*, the parameters, *β*, and covariates described in Table 1, *x*, is parameterised such that $\mu_{i}=exp(x_{i}\beta )$ [29], [30].

The number of appliance uses per week during the evening electricity peak originates from two data generating processes. Some unknown hurdle exists that distinguishes between households that use appliances in the evening peak period (i.e. $y_{i}=1,2,3,\;\ldots$) and those with no evening appliance uses (i.e. $y_{i}=0$). The log-likelihood for the two combined processes is

(2) L=ln(f(0))+{ln(1−f(0))+lnP(y)}

where f(0) represents the probability of the binary part of the model (i.e. probability of zero evening appliance uses) and P(y) represents the probability of a positive count. Modelling the binary hurdle with a logit specification and the count process, P(y), as a truncated negative binomial, the log-likelihood for negative binomial-logit hurdle model is [29]:

(3) $$L=\left\{ \begin{array}{cc} \text{if}\;(y=0), & \frac{1}{1+exp(x_{i}\beta )}\\ \text{if}\;(y>0) & y_{i}\;ln\left(\frac{exp(x_{i}\beta )}{1+\alpha \;exp(x_{i}\beta )}\right)-\frac{ln(1+\alpha \;exp(x_{i}\beta ))}{\alpha }+ln\;\Gamma \left(y_{i}+\frac{1}{\alpha }\right)\\ & -ln\;\Gamma \left(y_{i}+1\right)-ln\;\Gamma \left(\frac{1}{\alpha }\right)-ln(1-(1+exp(x_{i}\beta ))) \end{array}\right.$$

From a management or policy perspective a key item of interest is understanding how the count of appliance uses varies with household characteristics. As the mean of the negative binomial is given by *μ* and as all the explanatory variables are binary, the discrete marginal effect of a change in variable $x_{j}$ is

(4) $$\frac{\partial \mu }{\partial x_{j}}=\frac{\partial exp(x\beta )}{\partial x_{j}}=\mu \beta_{j}{|}_{x_{j}=1}-\mu \beta_{j}{|}_{x_{j}=0}$$

## 3 Results

All model estimates are reported in Table 2. First, considering the model on appliance uses across all periods of the day, the likelihood ratio test for the fitted model, at $\chi_{16}^{2}$=520.51, with a p-value <0.001 has explanatory power. However, just two attributes are chiefly associated with appliance use. The coefficient estimates on family size and number of appliances are all positive and statistically significant. Households with either more family members or a greater number of appliances (i.e. washing machine, dishwasher or clothes dryer) are likely to have a greater count of appliance uses per week than single person families, or those with one or no appliances. The coefficient estimate related to whether a person is at home during the day is not statistically significant, so having somebody be at home during the day is not associated with a higher (or lower) number of appliance use cycles. With the exception of one educational attainment variable none of the other socio-demographic variables are associated with the number of appliance uses.

**Table 2:** Model estimates: NB2 and NB2-logit hurdle models.

Dependent variable - appliance uses during:	all periods	evening peak	evening peak	
Model:	Negative binomial	Negative binomial	Negative binomial-logit hurdle	
	NB2	NB2	hurdle:	count:
			logit †	NB2 ‡
Education (ref: less than full secondary)				
Secondary level	-0.024	-0.050	0.981	-0.067
	(0.085)	(0.295)	(0.336)	(0.180)
Undergraduate level	-0.125	-0.203	1.077	-0.218
	(0.086)	(0.301)	(0.374)	(0.184)
Post-graduate level	-0.268***	-0.211	1.101	-0.199
	(0.095)	(0.322)	(0.416)	(0.199)
In employment	-0.057	0.298**	0.701**	0.055
	(0.044)	(0.151)	(0.122)	(0.094)
Age (ref: 18–34)				
Age 35–54	-0.015	-0.040	0.785	-0.130
	(0.049)	(0.164)	(0.153)	(0.098)
Age 55+	0.066	-0.031	0.782	-0.081
	(0.056)	(0.187)	(0.174)	(0.113)
Monthly income (ref: item non-response)				
€0–1999	-0.048	-0.013	1.039	-0.095
	(0.062)	(0.212)	(0.252)	(0.133)
€2,000-3999	0.043	0.091	1.245	0.180
	(0.057)	(0.190)	(0.28)	(0.114)
€4000+	0.063	0.166	1.168	0.232*
	(0.063)	(0.214)	(0.299)	(0.123)
Family size (ref: 1 person)				
2 persons	0.418***	0.717***	0.501***	0.370**
	(0.074)	(0.227)	(0.131)	(0.180)
3 persons	0.757***	1.069***	0.443***	0.719***
	(0.079)	(0.248)	(0.13)	(0.186)
4 persons	0.820***	1.190***	0.45***	0.883***
	(0.084)	(0.264)	(0.14)	(0.192)
5+ persons	1.074***	1.304***	0.351***	0.952***
	(0.087)	(0.286)	(0.119)	(0.199)
If person(s) usually at home during day	0.066	-0.462***	1.817**	-0.220**
	(0.054)	(0.172)	(0.378)	(0.098)
Number of appliances (ref: zero or 1 appliance)				
2 appliances	0.547***	0.592***	0.631**	0.404**
	(0.068)	(0.210)	(0.151)	(0.167)
3 appliances	0.985***	1.264***	0.263***	0.604***
	(0.066)	(0.208)	(0.063)	(0.161)
Constant	0.718***	-0.700	4.229	0.727**
	(0.133)	(0.434)	(2.131)	(0.298)
*α*	0.137***	2.601***		0.284***
	(0.104)	(0.081)		(0.047)
Observations	812	812	812	
Log-likelihood	-2160.52	-1486.26	-1406.35	
Akaike information criterion (AIC)	4357.046	3008.519	2882.69	
$\chi_{\left(16\right)}^{2}$	520.51	105.47	73.86	
p-value $\chi_{\left(16\right)}^{2}$	<0.001	<0.001	<0.001	

† Logit estimates reported as odds ratios & significance tests from 1.

‡ NB2 estimates reported as coefficients & significance tests from 0.

*** p<0.01, ** p<0.05, * p<0.1.

The second set of regression models in Table 2 relate to appliance use in the evening period, with results for both a standard negative binomial and negative binomial-logit hurdle model presented. Comparing the coefficient estimates for the count component across the two models, the signs of the coefficients match but there are substantial differences in their magnitudes. The negative binomial-logit hurdle model is the preferred model, as it respects the nature of the data generating process, but also because the relative likelihood of the negative binomial compared to the negative binomial-logit hurdle model based on the Akaike information criterion (AIC) is negligibly low [31].^1^ As is the case for the NB2 model for all time periods, increasing appliance use is associated with a higher number of family members and more appliances within the home. In contrast to the model for all time periods, having someone at home during the day impacts on evening appliance use. From the logit hurdle component of the model, households with someone at home during the day are 1.8 times more likely to have zero appliance uses during the evening peak period. Larger families, whether two or more persons, are less likely to have zero appliance uses during the evening period. A two-person family is half as likely as a single-person family to have zero appliance uses in the evening. For larger families the likelihood is even lower relative to a single-person family. As the number of considered appliances (i.e. washing machine, dishwasher or clothes dryer) increases within households, the likelihood of zero appliance uses during the evening period declines relative to households with just one or none of these three appliances.

The marginal impact on appliance uses cannot be interpreted directly from the coefficient estimates in Table 2 and instead are reported in Table 3 based on calculations using equation (4) and the delta method. First consider appliance uses during all periods. As the number of persons in the household increases, the combined number of washing machine, dishwasher and clothes dryer uses during a week increases. For a two compared to a one-person household, appliance uses across a week increases by two. For a five compared to a one-person household, there are an estimated 7 additional appliance uses. Mirroring the insignificant model coefficient on daytime occupancy, the marginal effect estimate associated with daytime occupancy is statistically insignificant. The marginal effect estimates associated with appliance ownership are slightly larger than those for family size. A household that owns all three of the considered appliances uses them an estimated 9 additional times during a week compared to a household with just one appliance.^2^

**Table 3:** Marginal Effects: Number of appliance uses.

Number of appliance uses per week:	all periods		during evening peak	
Model:	NB2		NB2-logit	
	marginal	p-value	marginal	p-value
	effect		effect	
Household members (ref: 1 person)				
2 persons	1.966***	<0.001	1.804**	0.050
3 persons	4.295***	<0.001	4.215***	0.002
4 persons	4.817***	<0.001	5.600***	<0.001
5 persons	7.305***	<0.001	6.524***	<0.001
Daytime occupancy (ref: no-one home)				
Person usually home during day	0.430	0.215	-1.083**	0.034
Number of appliances: washing machine, dishwasher or clothes dryer (ref: zero or 1)				
2 appliances	5.957***	<0.001	2.058**	0.027
3 appliances	9.228***	<0.001	2.673***	<0.001

*** p<0.01, ** p<0.05.

Turning to the evening period, the number of additional appliance uses per week is also increasing both in family size and in number of appliances owned. What is particularly noteworthy related to family size is that the marginal effect estimates for both all periods and the evening peak period are broadly similar; the 95% confidence intervals of the two point estimates at each family size category overlap. Earlier from the NB2 model we can say that larger families use the three appliances 2–7 additional times per week compared to single-person families. From the NB2-logit hurdle model we can deduce that these additional appliance uses occur during the evening peak period. During the evening peak period, families with two people or more use the three appliances 1.8–6.5 additional times per week compared to single-person families. When considering appliance ownership in the evening period, there are 2–2.6 additional appliance uses per week among families with 2–3 appliances relative to the reference category but these estimates are substantially lower than the comparable estimates for all periods. So unlike family size, the additional appliance uses associated with ownership of a greater number of appliances are not concentrated in the evening peak period. The outstanding estimate in Table 3 relates to daytime occupancy. Where there is a person normally at home during the day, the associated number of appliance uses during the evening peak is one use less than otherwise. As one might anticipate, when there is nobody usually at home during the day, household cleaning duties are concentrated in the other periods, which in most instances will be the evening time.

## 4 Discussion and conclusions

To efficiently deploy DSM mechanisms requires knowledge of curtailable loads and customers' preferences with respect to service interruption (i.e. deferred loads), around which there is extensive ongoing research. A particular challenge for developing reliable load profile data relates to domestic appliance ownership and use [17]. While there is strong understanding of the factors that systematically impact on aggregate residential loads [24], much less is known about the timing of residential loads, in general, and specifically appliance loads that can potentially be shifted to off-peak periods. Prior research has identified family structure/occupants; number of appliances; and daytime occupancy of the home as key determinants of residential loads [25], [26], [27]. The current research establishes that the same factors are closely associated with appliance loads during the evening peak but that the magnitude of the association differs, which has direct relevance for DSM policies.

The research also has relevance for customer load profile modelling. All three of the key factors systematically associated with peak period residential appliance use, and therefore loads, are factors that are not always readily observable to researchers or available in datasets commonly used in energy modelling. Energy system modelling, whether for DSM or other purposes, that utilises synthetic appliance load data should be based on models that incorporate these three factors: family size, daytime occupancy, and appliance ownership. Appliance load data based on archetypes related to building tenure, type or age; or socio-demographic variables such as income, age or education are likely to be inferior substitutes.

Prior research on domestic appliance use in Ireland suggests that appliance ownership and related energy use is associated with socio-demographic variables such as age, income, education or social status, as well as, building attributes [32], [33]. The current research finds no association between appliance use and occupants' age, income or education. The disparity between the earlier and current research on this matter may reflect the underlying data sources. The present study is based on survey data that specifically collects information on appliance ownership and use, whereas the earlier studies utilise data that is more wide ranging in its coverage, one being a household expenditure survey, and the other a survey on housing quality. O'Doherty et al. [32] acknowledge that the data they use is not sufficient to explain appliance usage and rather they use it to model the quantity of appliances present in households.

## Declarations

### Author contribution statement

**John Curtis:** Conceived and designed the experiments; Performed the experiments; Analyzed and interpreted the data; Contributed reagents, materials, analysis tools or data; Wrote the paper.

### Funding statement

This work was supported by the CREDENCE Project (Collaborative Research of Decentralisation, Electrification, Communications and Economics), a US-Ireland Research and Development Partnership Program funded by the 10.13039/100016337Department for the Economy Northern Ireland (USI 110), 10.13039/501100001602Science Foundation Ireland (16/US-C2C/3290) and The 10.13039/100000001National Science Foundation (0812121). Funding from the Economic and Social Research Institutés Energy Policy Research Centre is also gratefully acknowledged.

### Data availability statement

Data will be made available on request.

### Declaration of interests statement

The authors declare no conflict of interest.

### Additional information

No additional information is available for this paper.

## Footnotes

1 exp((2882.69-3008.519)/2) <0.0001.

2 The appliance ownership reference category is predominantly households with one appliance. Just 4% of households in the reference category own none of the three appliances.
